# BLIS study: a feasibility randomised controlled trial assessing compliance, acceptability and colonisation with different dosing regimens of the probiotic supplement Streptococcus salivarius K12 (Bactoblis) in adults in England

**DOI:** 10.1136/bmjopen-2026-120406

**Published:** 2026-09-15

**Authors:** Christopher R Wilcox, David W Cleary, Maclyn Augustine, Rebecca Anderson, Nick Francis, Mark Lown, Michael Moore, Paul Little, Stuart C Clarke, Merlin Willcox

**Affiliations:** 1 Primary Care Research Centre, University of Southampton School of Primary Care Population Sciences and Medical Education, Southampton, UK; 2 Department of Microbes, Infections, and Microbiomes, College of Medicine and Health & Institute of Microbiology and Infection, University of Birmingham, Birmingham, UK; 3 Institute of Microbiology and Infection, University of Birmingham, Birmingham, UK; 4 University Hospital Southampton NHS Foundation Trust, Southampton, UK; 5 University of Southampton Faculty of Medicine Health and Life Sciences, Southampton, UK; 6 NIHR Southampton Biomedical Research Centre, Southampton, UK; 7 Faculty of Medicine and Institute for Life Sciences, University of Southampton, Southampton, UK

**Keywords:** Clinical Trial, General Practice, INFECTIOUS DISEASES, MICROBIOLOGY, OTOLARYNGOLOGY, Feasibility Studies

## Abstract

**Objectives:**

*Streptococcus salivarius* K12 (SsK12) is a bacterium used as a probiotic with some evidence for preventing acute sore throat/tonsillitis, but the optimal dosing strategy is unclear. This study aimed to evaluate two dosing regimens of SsK12 to establish (a) the prevalence and duration of colonisation with SsK12 and (b) the acceptability and feasibility of these regimens.

**Design:**

A randomised, non-blinded feasibility trial.

**Setting:**

Primary care in the south of England.

**Participants:**

Adults with ≥2 episodes of sore throat within the 3 years before recruitment.

**Interventions:**

Participants were randomised to take SsK12 lozenges once weekly or daily over 14 days.

**Main outcome measures:**

The primary outcome was the prevalence and duration of colonisation with SsK12. This was determined by real-time PCR conducted on self-taken whole-mouth swabs provided by participants at baseline and on days 2, 7, 14, 21 and 35. Secondary outcomes were the compliance and acceptability of the two SsK12 dosing regimens, based on questionnaire data.

**Results:**

53 participants were recruited (26 randomised to weekly and 27 to daily dosing) between 26 April and 5 December 2023. All swabs were returned by 65.4% (17/26) and 70.4% (19/27) of the weekly and daily groups respectively. Swabs at all timepoints from participants with PCR positives observed at baseline (two and four from the weekly and daily group, respectively) were excluded. Therefore, swabs from 23 participants in the weekly and 22 in the daily group did not have evidence of baseline colonisation and were included in the microbiology analysis. SsK12 colonisation was found in 59.1% (13/22, 95% CI 38.6% to 79.6%) of samples in both groups on day 2; in 13.6% (3/22, 95% CI 0.0% to 28.0%) and 85.7% (18/21, 95% CI 70.8% to 100.0%) of the weekly and daily groups, respectively, on day 7; in 19.1% (4/21, 95% CI 2.3% to 35.8%) and 59.1% (13/22, 95% CI 38.6% to 79.6%), respectively, on day 14; and in only one participant (weekly group) on days 21 and 35. The dosing regimen was reported as easy to follow by 85.0% (17/20, 95% CI 69.4% to 100.0%) of the weekly and 96.2% (25/26, 95% CI 88.8% to 100.0%) of the daily dosing groups.

**Conclusions:**

SsK12 colonisation was more prevalent with daily dosing; however, colonisation was not maintained when dosing stopped. Both dosing regimens were acceptable to participants. These findings support the use of daily dosing in a future trial to evaluate the efficacy of SsK12 in preventing recurrence of sore throat/tonsillitis.

**Trial registration number:**

NCT04297878.

**STRENGTHS AND LIMITATIONS OF THIS STUDY**

- Participants with a history of recurrent sore throat were recruited—a cohort for whom prophylactic SsK12 may be most clinically relevant.
- Participants were randomised to dose regime to control for confounding, while those performing microbiological analysis were blinded to allocation, enhancing objectivity in the analysis of colonisation data.
- Participants were not blinded to group allocation, however, this is unlikely to have affected colonisation rates.
- Data regarding baseline characteristics, adherence, episodes of infection and antibiotic use during the study were self-reported by participants and therefore open to recall bias.
- Potential differences in sampling quality of self-taken swabs between participants and effects of transport and storage may have affected biomass available for analysis of colonisation.

## Introduction

 Acute sore throats are a common reason for inappropriate antibiotic prescriptions^1^ and are estimated to cost over £400 million annually in the UK due to consultations and lost productivity.^2^ Preventative options are needed. While most cases are viral, the most common causative bacteria are group A streptococci, typically *Streptococcus pyogenes*.^3 4^

Probiotic supplements may protect against infection,^5^ by improving mucosal barrier functions, inhibiting bacterial adhesion and growth, and modulating immune responses.^6^ The probiotic strain *Streptococcus salivarius K12* (SsK12) produces two class I lantibiotic bacteriocins (salivaricin A2 and salivaricin B),^7^ which are strongly antagonistic to *Streptococcus pyogenes* and partially antagonistic to *Moraxella catarrhalis, Haemophilus influenzae* and *Streptococcus pneumoniae*.^8 9^

There is preliminary evidence to support the prophylactic use of orally administered SsK12 against pharyngotonsillitis. However, most studies were conducted in children^10–19^ and many have high risk of bias and/or conflicts of interest.^20^ One clinical trial in adults, including 40 participants, reported a reduction in episodes of pharyngitis and/or tonsillitis in those given SsK12 daily for 90 days.^21^ This trial was non-randomised, unblinded, and had a conflict of interest. High-quality trials are therefore needed to clarify the clinical efficacy of SsK12.

There remains uncertainty about optimal dosing for colonisation of the oral cavity. Studies investigating the effect of daily SsK12 (with dosing periods varying from 3 days to 1 month) on colonisation of the oral cavity in adults suggest that daily dosing increases salivary SsK12 levels, but levels return to baseline when dosing stops.^22–26^ There is evidence that after daily dosing for 3 days, SsK12 levels remain high up to day eight before declining.^27^ Studies evaluating colonisation rates with less frequent dosing are required, as daily dosing may lead to reduced adherence over time and may be costly, even if clinically effective for preventing pharyngotonsillitis. Additionally, data on SsK12 colonisation in those with recurrent sore throat are lacking—further study is needed as prophylactic strategies are most clinically relevant for this group, and their oral microbiome may differ from that of healthy volunteers.

With a view to conducting an adequately powered randomised controlled efficacy trial in the future, this feasibility study aimed to evaluate two different dosing regimens of Ssk12 to establish (a) the prevalence and duration of colonisation with SsK12 and (b) the acceptability and feasibility of these two regimens.

## Methods

### Study design and setting

This prospective, non-blinded, feasibility randomised controlled trial compared two dosing regimens of SsK12 probiotic lozenges (Bactoblis). Participation was remote, with no face-to-face contact between participants and the research team. The study was registered on ClinicalTrials.gov (NCT04297878).

### Participant recruitment, inclusion and exclusion criteria

Five general practitioner (GP) practices in South England identified and invited all adult patients with two or more coded episodes in the past 3 years of either sore throat or tonsillitis, or prescriptions of phenoxymethylpenicillin. Individuals who contacted the study team underwent telephone screening to confirm eligibility, completed an online consent form, were posted a study pack and scheduled for a baseline study visit via video call. Inclusion criteria were adults aged ≥18 years with a history of two or more significant episodes of sore throat or tonsillitis (as judged by the participant) in the past 3 years. Exclusion criteria included active sore throat/tonsillitis, current smoking (or vaping), receipt of antibiotics or immunosuppressant medication within the last 30 days, and presence of a chronic disease/condition (such as those affecting the oropharynx) which might impact on colonisation, as judged by the study team.

### Randomisation

Participants were randomly assigned in a 1:1 allocation ratio to daily or weekly dosing. Random permuted blocks were generated by https://www.studyrandomizer.com. No stratification factors were used.

### Intervention

Bactoblis is a strawberry-flavoured lozenge that dissolves in 3–5 minutes in the mouth. Bactoblis is produced commercially for European Union sale according to the standards of GMP, HACCP and ISO22000. Each lozenge contains >5 billion × 10^9^ cfu of SsK12 in a lyoprotectant matrix of trehalose, lactitol and maltodextrin, isomaltulose, flavour and processing aids. The weekly group took two lozenges at night on days 1, 7 and 14 while the daily group took one lozenge at night on days 1–14. Participants were instructed to brush their teeth prior to taking the lozenges as per the manufacturer’s guidelines.

### Sample size

Previous evidence suggests that up to 86% of participants have evidence of SsK12 colonisation at day 7 after 3 days of daily oral SsK12 administration,^23^ while there is no prior evidence related to weekly administration. Therefore, assuming that 75% of people in the weekly group would show evidence of colonisation at day 7, and we wished to demonstrate that continuous treatment in the daily group is 15% better (ie, 90% colonised), then for 90% power, 21 participants per group would be required. If only 60% of the weekly group were colonised at day 7, and at least 75% of the daily group colonised, then for 90% power one would need 32 people per group. Given the uncertainty over colonisation rates due to lack of previous research in this area, we opted for 25 per group.

### Data collection

Participants were instructed to take two whole-mouth swabs (by swabbing the inside of both cheeks, the back of the tongue and the soft palate) in the morning on days 0, 2, 7, 14, 21 and 35, and to post these on the day of collection. Online questionnaires, completed on days 15 and 35, asked participants to report adherence; views on the palatability of the lozenges, the dosing regimen, ease of swab-taking and postal return; potential side-effects; infectious illnesses and any antibiotics taken during the study. Open questions allowed participants to express any additional issues.

### Laboratory procedures

Swabs were analysed at the microbiology facilities of the Infectious Disease Epidemiology Group, Faculty of Medicine at the University of Southampton. Molecular analysis was conducted using real-time, quantitative PCR to detect the presence of SsK12. DNA was extracted from swabs using commercially available QIAamp DNA Mini kits (Qiagen, UK). Real-time PCRs used three targets: *sboA*, *sboG* and *sboK*, using previously published primers and PCR cycling conditions.^28^ The *sboA* assay was run in TaqManÔ format, with the other two as SYBR green assays using Luna Universal Probe qPCR Master Mix (NEB, UK) and Quantinova SYBR green PCR kits (Qiagen, UK), respectively. Assays were run on a Rotorgene Q (Qiagen, UK) as triplicates per sample. Quantification was achieved by comparison of cycle-threshold (*Ct*) values to those of a standard curve generated using genomic DNA of SsK12. Colonisation with SsK12 was defined by a positive PCR for any one of three gene target assays (*sboA*, *sboG* or *sboK*), with a *Ct* value of ≤35. Microbiologists were blinded to allocation.

### Outcomes and statistical analysis

The primary outcome was the prevalence and duration of colonisation with SsK12. Secondary outcomes were the compliance and acceptability of the two SsK12 dosing regimens. Statistical analysis was performed using STATA V.18 (StataCorp LLC). As this was a feasibility study, only descriptive statistics are reported. Continuous variables are reported using the median and IQR. Categorical variables are reported as percentages, with corresponding 95% CIs when reporting results according to group.

### Patient and public involvement

Patients and the public were not involved in the design of this feasibility study.

## Results

### Recruitment and adherence

**Figure 1:**
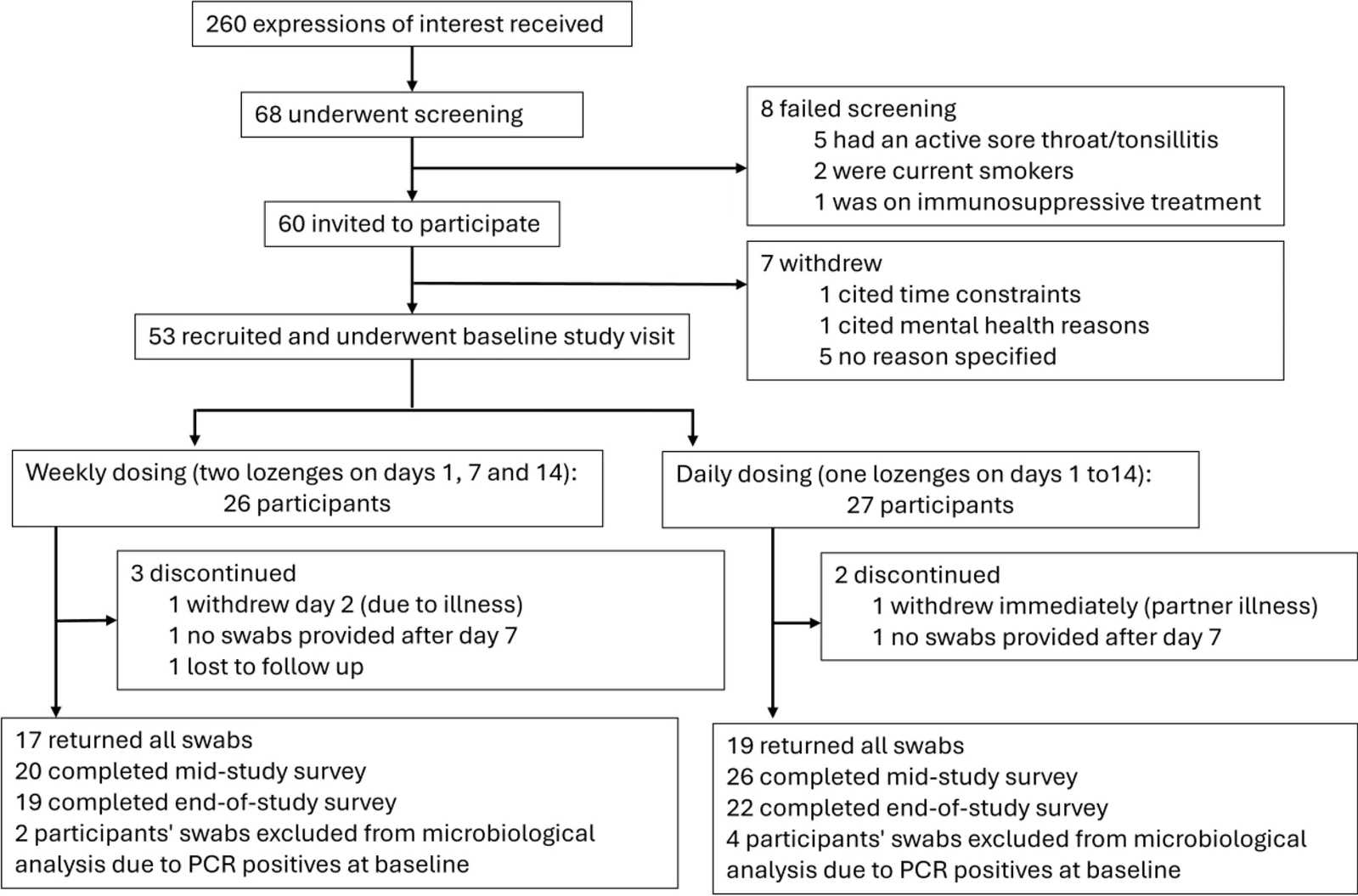
CONSORT diagram displaying flow of participants. CONSORT, Consolidated Standards of Reporting Trials.

Between 26 April 2023 and 5 December 2023, 68 individuals were screened and 77.9% (53/68) recruited to the study (26 randomised to weekly and 27 to daily dosing). The flow of participants is displayed in figure 1. Participants in both groups were similar in terms of age and gender distribution, while adherence to the dosing regimen was high with no difference between groups, and most participants completed the study questionnaires (table 1).

**Table 1:** Summary of participant characteristics and adherence to study procedures

	Weekly dosing	Daily dosing	All participants
Age, median (IQR)	38.5 years (IQR 31.3−46.3)	36.0 years (IQR 33.0–49.0)	37.0 years (IQR 31.8–47.5)
Sex, % female (n), 95% CI	80.8% (21/26), 95% CI 65.6% to 95.9%	81.5% (22/27), 95% CI 66.8% to 96.1%	81.1% (43/53), 95% CI 70.6% to 91.7%
Mid-study questionnaire completed, % (n), 95% CI	76.9% (20/26), 95% CI 60.7% to 93.1%	96.3% (26/27), 95% CI 89.2% to 100.0%	86.8% (46/53), 95% CI 77.7% to 95.9%
End-of-study questionnaire completed, % (n), 95% CI	73.1% (19/26), 95% CI 56.0% to 90.1%	81.5% (22/27), 95% CI 66.8% to 96.1%	77.4% (41/53), 95% CI 66.1% to 88.6%
Full adherence to probiotic dosing schedule, % (n), 95% CI*	85.0% (17/20), 95% CI 69.4% to 100.0%	88.5% (23/26), 95% CI 76.2% to 100.0%	87.0% (40/46), 95% CI 77.2% to 96.7%
All swabs returned, % (n), 95% CI	65.4% (17/26), 95% CI 47.1% to 83.4%	70.4% (19/27), 95% CI 53.2% to 87.6%	67.9% (36/53), 95% CI 55.4% to 80.5%

* The denominator for full adherence is the number of participants who completed the mid-study questionnaire.

### Swab return

At least one swab was returned by 96.2% (51/53), as one participant withdrew from the study (due to family illness) and one was lost to follow-up. All six sets of study swabs were returned by 65.4% (17/26) of the weekly and 70.4% (19/27) of the daily-dosing groups, respectively. There was a median delay of 2 days (IQR 1–4 days) between swabs being taken and received in the lab.

### Colonisation

**Figure 2:**
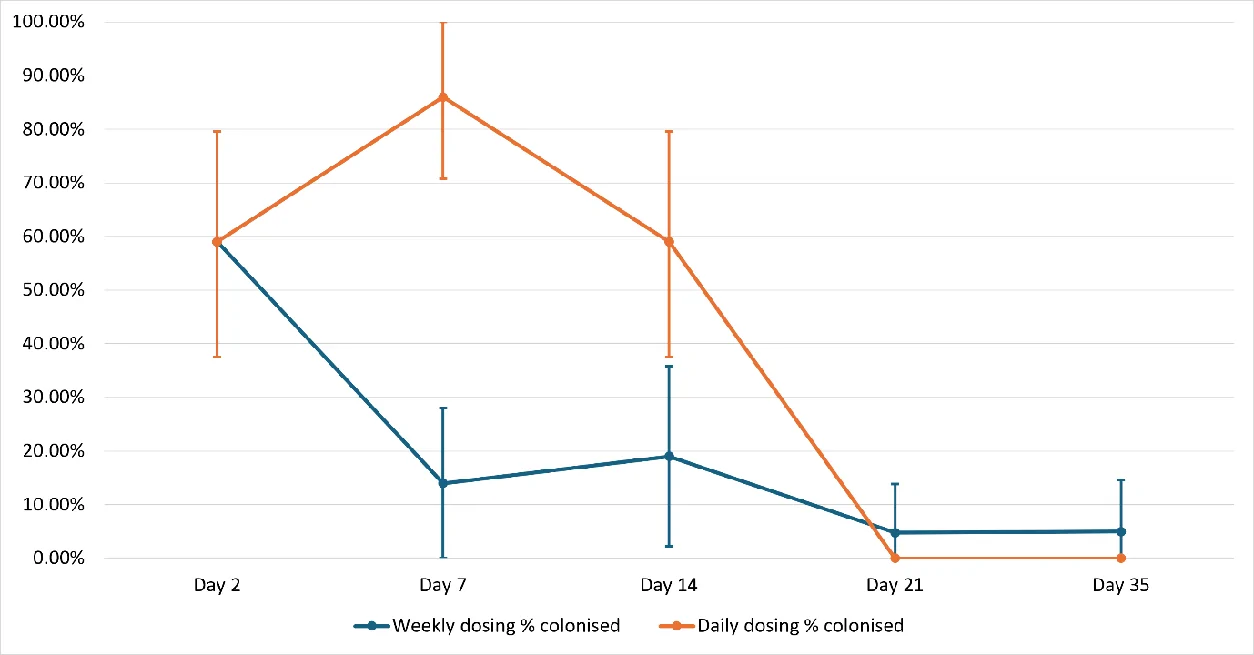
Comparison of prevalence of colonisation between the daily and weekly dosing groups. Vertical lines represent 95% CIs.

Swabs at all timepoints from six participants (two and four from the weekly and daily groups, respectively) were excluded due to PCR positives observed at baseline. Therefore, swabs from 23 participants in the weekly and 22 in the daily group did not have evidence of baseline colonisation and were included in the microbiology analysis. During the dosing period, there was evidence of colonisation in samples from both groups, but colonisation rates were higher in the daily-dosing group (figure 2 and table 2). After the dosing period ended, there was minimal evidence of colonisation in the weekly-dosing group, and no evidence of colonisation in the daily-dosing group.

**Table 2:** Comparison of prevalence of colonisation with SsK12 between weekly versus daily dosing

	Day 2	Day 7	Day 14	Day 21	Day 35
Weekly dosing, % positive for SsK12 (n), 95% CI	59.1% (13/22), 95% CI 38.6% to 79.6%	13.6% (3/22), 95% CI 0.0% to 28.0%	19.1% (4/21), 95% CI 2.3% to 35.8%	4.8% (1/21), 95% CI 0.00% to 13.9%	5.0% (1/20), 95% CI 0.0% to 14.6%
Daily dosing, % positive for SsK12 (n), 95% CI	59.1% (13/22), 95% CI 38.6% to 79.6%	85.7% (18/21), 95% CI 70.8% to 100.0%	59.1% (13/22), 95% CI 38.6% to 79.6%	0.0% (0/19)	0.0% (0/18)
Both groups, % positive for SsK12 (n), 95% CI	59.1% (26/44), 95% CI 44.6% to 73.6%	48.8% (21/43), 95% CI 33.9% to 63.8%	39.5% (17/43), 95% CI 24.9% to 54.2%	2.5% (1/40), 95% CI 0.0% to 7.3%	2.6% (1/38), 95% CI 0.0% to 7.7%

### Views on the probiotic, dosing regimen and self-swabbing

Of those who completed the mid-study questionnaire, most agreed that the lozenges had a pleasant taste (table 3).

**Table 3:** Views on taste of the probiotic and ease of following the dosing regimen reported by participants who completed the mid-study questionnaire

	Strongly agree	Agree	Neutral	Disagree	Strongly disagree
Taste of lozenges was pleasant, % (n), 95% CI	30.4% (14/46), 95% CI 17.1% to 43.7%	37.0% (17/46), 95% CI 23.0% to 50.9%	30.4% (14/46), 95% CI 17.1% to 43.7%	2.2% (1/46), 95% CI 0.0% to 6.4%	0.0% (0/46)
Dosing regimen was easy to follow, % (n), 95% CI	Both groups: 60.9% (28/46), 95% CI 46.8% to 75.0% Weekly dosing: 45.0% (9/20), 95% CI 23.2% to 66.8% Daily dosing: 73.1% (19/26), 95% CI 56.0% to 90.1%	Both groups: 30.4% (14/46), 95% CI 17.1% to 43.7% Weekly dosing: 40.0% (8/20), 95% CI 18.5% to 61.5% Daily dosing: 23.1% (6/26), 95% CI 6.9% to 39.3%	Both groups: 6.5% (3/46), 95% CI 0.0% to 13.7% Weekly dosing: 10.0% (2/20), 95% CI 0.0% to 23.2% Daily dosing: 3.9% (1/26), 95% CI 0.0% to 11.2%	Both groups: 2.2% (1/46), 95% CI 0.0% to 6.4% Weekly dosing: 5.0% (1/20), 95% CI 0.0% to 14.6% Daily dosing: 0.0% (0/26)	Both groups: 0.0% (0/46) Weekly dosing: 0.0%, (0/20) Daily dosing: 0.0% (0/26)

Potential side effects were reported by 21.7% (10/46) of the mid-study questionnaire respondents, of whom 70.0% were from the daily group (table 4). Six reported a sore throat (three alongside cough and/or coryzal symptoms), one reported a dry mouth, one reported a change in vaginal discharge, one reported abdominal bloating, and one reported new mouth ulcers. No additional concerns were raised in the end-of-study questionnaire. Over 90% of participants in both groups felt that self-taken swabs were easy to take, package and post (table 5).

**Table 4:** Summary of all the potential side effects reported by participants who completed the mid-study questionnaire

	Both groups	Weekly dosing	Daily dosing
Any side effect reported, % (n), 95% CI	21.7% (10/46), 95% CI 9.8% to 33.7%	15.0% (3/20), 95% CI 0.0% to 30.7%	26.9% (7/26), 95% CI 9.9% to 44.0%
Sore throat with cough and/or coryza, % (n), 95% CI	4.4% (2/46), 95% CI 0.0% to 10.2%	5.0% (1/20), 95% CI 0.0% to 14.6%	3.9% (1/26), 95% CI 0.0% to 11.2%
Sore throat without cough or coryza, % (n), 95% CI	6.5% (3/46), 95% CI 0.0% to 13.7%	5.0% (1/20), 95% CI 0.0% to 14.6%	7.7% (2/26), 95% CI 0.0% to 17.9%
Cough and coryza without sore throat, % (n), 95% CI	2.2% (1/46), 95% CI 0.0% to 6.4%	0.0% (0/20)	3.6% (1/26), 95% CI 0.0% to 11.2%
Dry mouth, % (n), 95% CI	2.2% (1/46), 95% CI 0.0% to 6.4%	5.0% (1/20), 95% CI 0.0% to 14.6%	0.0% (0/26)
Mouth ulcers, % (n), 95% CI	2.2% (1/46), 95% CI 0.0% to 6.4%	0.0% (0/20)	3.6% (1/26), 95% CI 0.0% to 11.2%
Change in vaginal discharge, % (n), 95% CI	2.2% (1/46), 95% CI 0.0% to 6.4%	0.0% (0/20)	3.9% (1/26), 95% CI 0.0% to 11.2%
Abdominal bloating, % (n), 95% CI	2.2% (1/46), 95% CI 0.0% to 6.3%	0.0% (0/20)	3.9% (1/26), 95% CI 0.0% to 11.2%

**Table 5:** Views on the process of self-swabbing and posting the swabs reported by participants who completed the mid-study questionnaire

	Strongly agree	Agree	Neutral	Disagree	Strongly disagree
Self-taken swabs were easy to perform, % (n)	43.5% (20/46)	50.0% (23/46)	2.2% (1/46)	4.4% (2/46)	0.0% (0/46)
Self-taken swabs were easy to package and post, % (n)	50.0% (23/46)	47.8% (22/46)	0.0% (0/46)	2.2% (1/46)	0.0% (0/46)

### Respiratory tract infections and antibiotic use

Of those who completed the end-of-study questionnaire, 47.4% (9/19) and 27.3% (6/22) reported having a respiratory tract infection in the weekly and daily groups, respectively (table 6).

**Table 6:** Respiratory tract infections reported by participants who completed the end-of-study questionnaire

	Both groups	Weekly dosing	Daily dosing
Any respiratory tract infection, % (n), 95% CI	36.6% (15/41), 95% CI 21.8% to 51.3%	47.4% (9/19), 95% CI 24.9% to 69.8%	27.3% (6/22), 95% CI 8.7% to 45.9%
Sore throat and coryza, % (n), 95% CI	7.3% (3/41), 95% CI 0.0% to 15.3%	10.5% (2/19), 95% CI 0.0% to 24.3%	4.5% (1/22), 95% CI 0.0% to 13.3%
Sore throat without additional symptoms, % (n), 95% CI	17.1% (7/41), 95% CI 5.6% to 28.6%	26.3% (5/19), 95% CI 6.5% to 46.1%	9.1% (2/22), 95% CI 0.0% to 21.1%
Coryza without additional symptoms, % (n), 95% CI	7.3% (3/41), 95% CI 0.0% to 15.3%	10.5% (2/19), 95% CI 0.0% to 24.3%	4.6% (1/22), 95% CI 0.0% to 13.3%
Cough, % (n), 95% CI	2.4% (1/41), 95% CI 0.0% to 7.2%	0.0% (0/19)	4.6% (1/22), 95% CI 0.0% to 13.3%
Sinusitis, % (n), 95% CI	2.4% (1/41), 95% CI 0.0% to 7.2%	0.0% (0/19)	4.6% (1/22), 95% CI 0.0% to 13.3%

One participant from the weekly group reported taking a penicillin antibiotic but did not provide dates. Two participants from the daily group reported taking antibiotics during the study, one of whom took two 3-day courses of nitrofurantoin (separated by 2 days) for a urinary tract infection during the first week of the study, and the other participant provided no further details.

## Discussion

### Summary of key results

Our study showed that it was feasible to remotely recruit and follow-up participants based on a history of recurrent sore throat in their GP records. It was feasible to ask them to self-administer and return mouth swabs in the post, and to complete study questionnaires online. Adherence rates were high, and the probiotic was well-tolerated with no serious side effects.

To the best of our knowledge, this is the first study to report SsK12 colonisation data in adults with a history of recurrent sore throat, and to compare weekly versus daily administration of SsK12. Our findings suggest that daily dosing is more effective than weekly dosing at achieving and maintaining colonisation, however there was some evidence of SsK12 colonisation with weekly administration. In future trials it would make sense to use daily dosing. In both groups, there was minimal evidence of colonisation when dosing had stopped, suggesting that ongoing dosing is required to maintain colonisation. The colonisation rate in the daily dosing group was higher on day seven (85.7% of samples positive) than day 14 (59.1% of samples positive), possibly due to reduced compliance in the second week, as one participant missed doses on days 10 and 12 while another participant missed two doses but did not recall on which days.

Overall, no serious adverse effects were reported, supporting the short-term safety of prophylactic SsK12 lozenges. Although 21.7% (10/46 participants) reported potential side effects, the cases of sore throat may have been due to respiratory tract infection rather than a direct effect of the lozenges, as 10 participants reported experiencing a respiratory tract infection during the study.

### Comparison with existing literature

Previous studies in adults^22 23 25 26^ administered SsK12 daily, with the dosing period varying from 3 days to 1 month, and data suggest that maintaining colonisation is dependent on ongoing SsK12 dosing—consistent with our findings. One of these studies^23^ reported the proportion of participants with evidence of SsK12 colonisation (after a loading regimen of 3 days of SsK12) at similar timepoints (days 3, 7, 14 and 28) to our study, and colonisation rates from saliva samples appeared to be better maintained (93% on day 3, 86% on day 7, 64% on day 14 and 28.6% on day 28) compared with the daily dosing group in our study. Differences in sampling methods may explain this, as unlike in our study, participants used a chlorhexidine mouthwash before administering SsK12. Furthermore, we analysed self-taken whole-mouth swabs, not saliva samples, and the amount of biomass available may have differed between participants based on the quality of self-swabbing.

Our findings also support the short-term safety of SsK12. Similar studies in adults have also reported no serious adverse effects associated with daily SsK12 use.^21–23^ One study reported a case of hives which resolved on discontinuation; however, this study used a multiorganism probiotic which included other organisms alongside SsK12,^25^ whereas we used a single-strain probiotic.

### Strengths

Participants were randomised to dose regime to control for confounding. Adherence to both dosing regimens was good and the completion rate of study questionnaires was high. Furthermore, those performing microbiological analysis were blinded to allocation, enhancing objectivity in the analysis of colonisation data. We included participants with a history of recurrent sore throat—a cohort for whom prophylactic SsK12 is most clinically relevant. This study has also, for the first time, evaluated a weekly dosing regimen in terms of acceptability to participants and associated colonisation rates.

### Limitations

Participants were not blinded to group allocation, however this is unlikely to have affected colonisation rates. Second, data regarding baseline characteristics was self-reported by participants, including information on history of sore throats. Participants with a history of viral, rather than bacterial sore throat may have therefore been included. Similarly, data on adherence, episodes of infection and antibiotic use during the study were also self-reported and therefore open to recall bias. Finally, microbiology samples were self-taken swabs and were posted to the laboratory. The extent to which the quality of sampling differed between participants, alongside the potential effects of transport and storage on biomass available for analysis may have impacted the colonisation data. However, this study was designed during the COVID-19 pandemic and remote data collection was therefore preferable; additionally, there is evidence that self-swabbing may be as effective as healthcare professional swabs in detecting bacteria in the respiratory tract.^29^ Furthermore, participants may have been more comfortable with self-swabbing given the requirement for COVID-19 testing during periods of restrictions.

### Implications for future research

The effect of SsK12 supplementation on the overall oral microbiome is uncertain. One study in young children^24^ and two studies in adults with limited sample sizes^23 25^ reported no significant changes to the salivary microbiome associated with daily SsK12 administration with dosing periods up to 30 days. Further investigation after consistent dosing over a prolonged period may reveal different results.

The efficacy of SsK12 for preventing sore throat/tonsillitis is uncertain, due to the high risk of bias and/or conflict of interest associated with most previous studies. Our findings support the feasibility of an adequately powered randomised, placebo-controlled trial, which is crucial for robustly investigating the clinical efficacy of daily prophylactic SsK12. Preventive dosing at the onset of symptoms was found effective with nasal sprays to reduce symptoms of upper-respiratory tract infections^30^. It would be useful to investigate whether administering SsK12 at symptom onset also reduces length of symptoms. If proven to be clinically effective, this is likely to be more cost-effective than long-term daily prophylaxis.

## Conclusions

SsK12 colonisation was more prevalent with daily dosing; however, colonisation was not maintained when dosing stopped. Both weekly and daily dosing with SsK12 lozenges was acceptable to participants. These findings support the feasibility of a future fully powered efficacy trial, which is required for robustly investigating whether daily SsK12 can prevent recurrence of sore throat/tonsillitis.

## Data Availability

Data are available on reasonable request.
