# Supplementary material for: BLIS study: a feasibility randomised controlled trial assessing compliance, acceptability and colonisation with different dosing regimens of the probiotic supplement Streptococcus salivarius K12 (Bactoblis) in adults in England
Source: BMJ Open. 2026 Sep 15;16(9):e120406. doi: 10.1136/bmjopen-2026-120406 (PMC13583812; doi:10.1136/bmjopen-2026-120406)
Supplement: Supplementary file 1 [file bmjopen-2026-120406.reviewer_comments.pdf]

PEER REVIEW HISTORY

BMJ Open publishes all reviews undertaken for accepted manuscripts. Reviewers are asked to complete a checklist review form and are provided with free text boxes to elaborate on their assessment. These free text comments are reproduced below.

ARTICLE DETAILS

Title (Provisional)

The BLIS study: a feasibility randomised controlled trial assessing compliance, acceptability and colonisation with different dosing regimens of the probiotic supplement Streptococcus salivarius K12 (Bactoblis®) in adults in England

Authors

Wilcox, Christopher R; Cleary, David W; Augustine, Maclyn; Anderson, Rebecca; Francis, Nick; Lown, Mark; Moore, Michael; Little, Paul; Clarke, Stuart C; Willcox, Merlin

---

VERSION 1 - REVIEW

**Reviewer**	**1**
**Name**	**Thornley, Simon**
**Affiliation**	**The University of Auckland**
**Date**	**07-May-2026**
**COI**	**None**

The study has modest aims, but is clearly and succinctly presented and interpreted. My only suggestion is that a formal statistical test for differences between the two groups be conducted, since the study is randomised, particularly for the key outcome of colonisation.

I also think the results would be better communicated to an audience with a visualisation rather than table. Otherwise, I think the manuscript is well written and logically presented.

**Reviewer**	**2**
**Name**	**Rai, Junu**
**Affiliation**	**Tribhuvan University Institute of Medicine, Clinical Microbiology**
**Date**	**19-Jun-2026**
**COI**	**None**

Good research as well as writing.

Some area that require minor upgrade are:

1. Uniformity of data presentation such as decimal placements in numerical data.

2. Tabular presentation might need some clarity. Graphical presentation can be considered especially when showing the longitudinal data presentation.

**Reviewer**	**3**
**Name**	**Daniels, Rob**
**Affiliation**	**University of Exeter**
**Date**	**13-Jul-2026**
**COI**	**None**

This is an interesting project with outcomes that are relevant to a common primary care problem. The paper is well written and clear in its aims, methodology and results and the methodology is appropriate for the trial. Strengths and limitations are discussed in detail and overall the paper is helpful in determining optimum dosing strategy.

VERSION 1 - AUTHOR RESPONSE

Reviewer: 1
Dr. Simon Thornley, The University of Auckland
Comments to the Author:
The study has modest aims, but is clearly and succinctly presented and interpreted. My only suggestion is that a formal statistical test for differences between the two groups be conducted, since the study is randomised, particularly for the key outcome of colonisation.

Thank you for taking the time to review our manuscript and for your positive feedback and comments. This was a feasibility study designed predominantly to provide preliminary data to inform future trials. The study did not set out to formally test a hypothesis and therefore we do not feel that it would be appropriate to include formal statistical hypothesis testing. We have reported confidence intervals and feel that this is the most appropriate way of reporting uncertainty related to random variation in a study of this type.

I also think the results would be better communicated to an audience with a visualisation rather than table. Otherwise, I think the manuscript is well written and logically presented.

Thank you for your positive feedback, and for your suggestion on how we could better communicate our findings. We have added a graph (Figure 2) to present the longitudinal data comparing prevalence of colonisation between the daily and weekly dosing groups.

Reviewer: 2
Dr. Junu Rai, Tribhuvan University Institute of Medicine
Comments to the Author:
Good research as well as writing.
Some area that require minor upgrade are:
1. Uniformity of data presentation such as decimal placements in numerical data.

Many thanks for your comment. We have updated the manuscript to ensure that when reporting our results, all numerical data are consistently reported to 1 decimal place in the text and tables. We noticed that in very few instances, the proportion rather than the percentage was reported; we have edited this to ensure percentages are reported consistently throughout, and these changes are highlighted in yellow as the numbers differ slightly when compared to the original submission file.

2. Tabular presentation might need some clarity. Graphical presentation can be considered especially when showing the longitudinal data presentation.

Thank you for your suggestion on how we could better communicate our findings. We have added a graph (Figure 2) to present the longitudinal data comparing prevalence of colonisation between the daily and weekly dosing groups.

Reviewer: 3
Dr. Rob Daniels, University of Exeter
Comments to the Author:
This is an interesting project with outcomes that are relevant to a common primary care problem. The paper is well written and clear in its aims, methodology and results and the methodology is appropriate for the trial. Strengths and limitations are discussed in detail and overall the paper is helpful in determining optimum dosing strategy.

Many thanks for taking the time to review our manuscript and for providing this positive feedback
